# Acute Hepatitis B with Pancreatitis and Cholecystitis Leading to Acute Liver Failure and Death

**DOI:** 10.5811/cpcem.2018.7.38344

**Published:** 2018-08-15

**Authors:** Andrew R. Albert, Ronald Valencia, Janet A. Smereck

**Affiliations:** * University of Pennsylvania, Department of Emergency Medicine, Philadelphia, Pennsylvania; † Georgetown University Hospital, Department of Emergency Medicine, Washington, District of Columbia

## Abstract

Acute liver failure is defined as severe acute liver injury, concurrent with encephalopathy and loss of hepatic synthetic function, in a patient without known pre-existing liver disease. Evaluation of acute liver failure in the emergency department should focus on identification of treatable causes. Acute liver failure from acute hepatitis B infection is a rare but potentially lethal occurrence. Multi-organ dysfunction from acute liver failure may be exacerbated by metabolic and inflammatory reactions associated with acute pancreatitis, which accompanies approximately 5% of cases of acute viral hepatitis. Transplant-free survival rate with liver failure from acute hepatitis B is unfortunately less than 20%.

## INTRODUCTION

The critically ill patient presenting with fever, abdominal pain, and jaundice represents a diagnostic and therapeutic challenge in the emergency department (ED). Acute hepatitis B causing acute liver failure is a rare occurrence; multi-organ complications of acute liver failure are associated with high case fatality, despite advances in etiology-specific therapies and availability of urgent liver transplantation.^1^ Pancreatitis may complicate up to 35% of acute liver failure cases. Acute viral hepatitis is associated with pancreatitis and cholecystitis in about 5% of cases, due to direct tissue penetration by the virus itself as opposed to ductal obstruction. 2 Although pancreatitis associated with viral hepatitis generally follows a benign course, multisystem failure with hepatic encephalopathy in the setting of acute liver failure may be exacerbated by impaired metabolism and inflammatory cytokine reactions that accompany acute pancreatitis.^2^–^4^

## CASE REPORT

A 39-year-old man with no known health issues prior to ED presentation,was brought to the ED by family members with concerns for fever, generalized weakness, and abdominal pain of one week’s duration. He had been evaluated at an urgent care facility and referred to the ED after treatment with acetaminophen for temperature of 39.2 °C and ondansetron for nausea; rapid antigen testing for influenza and streptococcus were negative. He reported one week of fevers, night sweats, and anorexia with non-radiating, right upper quadrant pain, as well as multiple episodes of vomiting and the development of loose, gray-colored stools. He denied back pain or urinary symptoms. He had taken a small number of acetaminophen tablets of unknown dosage over the preceding week for fever, but denied regular or excessive use. He denied recent travel, unusual foods, herb or mushroom ingestion, ethanol use, or intravenous (IV) drug abuse. Socially, he admitted to daily marijuana and tobacco use.

Physical examination revealed an acutely ill man with scleral icterus, who was diaphoretic and moaning, complaining of pain. Vital signs on presentation included an oral temperature of 37.3 °C, heart rate of 78 beats per minute, blood pressure of 132/70 millimeters of mercury, and respiratory rate of 20 breaths per minute. Mucous membranes were dry, lungs were clear, and the heart sounds were regular without murmurs or gallops. The abdomen was soft with moderate tenderness in the right upper quadrant without guarding. The liver edge was palpable two centimeters (cm) inferior to the costal margin in the midclavicular line. He was slow to answer but oriented to person, place and time.

Laboratory findings were notable for significantly elevated white blood cell count of 22,300 / millimeters cubed (mm^3^), hemoglobin of 18.7 grams per deciliter (g/dL), platelets of 171,000 /mm.^3^ Liver function studies were remarkable for total bilirubin of 16.4 milligrams per deciliter (mg/dL), direct bilirubin of 10.6 mg/dL, aspartate aminotransferase of 2,682 international units per liter (IU/L), alanine aminotransferase of 7,521 IU/L, and alkaline phosphatase of 288 IU/L. Lipase was markedly elevated at 16,879 IU/L. Serum bicarbonate was 24 milliequivalents per liter (mEq/L), blood urea nitrogen was 26 mg/dL, and creatinine 1.53 mg/dL. Glucose was 61 mg/dL, and the remainder of electrolyte panel was normal. Coagulation studies revealed prothrombin time of 41.1 seconds and international normalized ratio (INR) of 4.3. Venous blood gas was notable for pH of 7.38 with lactate significantly elevated at 12.7 mEq/L. Toxicology workup was negative: ethanol less than 3 mg/dL and acetaminophen less than 2 micrograms per liter (μg/mL). Urine screen for drugs of abuse was positive for cannabinoids but negative for benzodiazepines, phencyclidine, opiates, and amphetamines.

Initial imaging included a right upper quadrant ultrasound (US) that revealed gallbladder distension with sludge, peri-cholecystic fluid and anterior wall thickening, but no gallstones. The common bile duct was measured at five millimeters (mm). Computerized tomography (CT) of the abdomen and pelvis obtained without contrast confirmed abnormal gallbladder findings from US and also demonstrated an edematous pancreas with peri-pancreatic fat stranding, without obstructing mass, as well as trace ascites. Initial management included a bolus of two L of IV crystalloids as well as broad-spectrum antibiotic coverage for possible biliary sepsis (1.5 grams [g] vancomycin and 3.375g piperacillin-tazobactam). A repeat bedside glucose of 53 mg/dL was treated with IV dextrose.

The patient was admitted to the medical intensive care unit with consults from the surgical and gastroenterology services. Primary admission diagnoses included acute hepatitis, acalculous cholecystitis and acute pancreatitis of unclear etiology. Lipid panel, including triglycerides, returned normal and the pattern of liver function abnormality did not point to an obstructive picture. Hepatitis serologies subsequently returned positive and suggestive of acute infection: hepatitis B core immunoglobulin M (IgM) reactive, hepatitis B surface antigen reactive, hepatitis B e-antigen and antibody reactive, and hepatitis B deoxyribonucleic acid level significantly elevated at 51,416 IU/mL, indicating highly active viral replication. Testing for cytomegalovirus, hepatitis C virus, hepatitis A virus, hepatitis D virus, hepatitis E virus, Epstein-Barr virus, and human immunodeficiency virus 1 and 2 were all negative.

The patient was questioned regarding risk factors for viral hepatitis; he stated he was sexually active with male and female partners and admitted to unprotected oral intercourse three weeks prior to his acute illness and barrier-protected anal intercourse six months prior to presentation. There was no history of tattoos or blood transfusions and no known prior history of hepatitis infection.

**CPC-EM Capsule**

What do we already know about this clinical entity?

Acute liver failure from acute hepatitis B virus is rare, but potentially lethal; case fatalities are associated with encephalopathy and cerebral edema.

What makes this presentation of disease reportable?

A rapidly progressive case of acute hepatitis B infection, associated with pancreatitis and cholecystitis, was refractory to aggressive therapeutic measures.

What is the major learning point?

Acute hepatitis B must be considered when determining the etiology of acute liver failure; early directed therapies in a transplantation center may improve survival.

How might this improve emergency medicine practice?

Although rare, acute hepatitis B may present with acute liver failure, with a high case fatality, necessitating specific therapeutic measures to improve survival.

Antiviral treatment was initiated with IV entecavir; IV *N*-acetylcysteine therapy was given for hepatoprotective effects. Antimicrobial therapy was continued with piperacillin-tazobactam, although cultures of blood and urine showed no growth on hospital day 2, and remained negative. Consultation with transplant surgery placed the patient on emergent status for priority liver transplantation.

The patient developed acute mental status changes with drowsiness alternating with agitation; he could no longer speak in full sentences, and was not oriented to time or situation. Serum glucose was 153 mg/dL and CT of the brain did not reveal structural abnormalities. Progressive decline in level of consciousness with asterixis was associated with ammonia level of 228 micromoles per liter (μmol/L); hepatic encephalopathy was treated with lactulose and rifaximin, given orally. IV vitamin K1 and fresh frozen plasma were given for treatment of worsening coagulopathy, with INR increased to 9.0. He was intubated for airway protection after a vomiting episode with possible aspiration.

The patient’s mental status continued to worsen, and repeat unenhanced head CT demonstrated interval development of cerebral edema with early signs of brainstem herniation. After neurosurgery consultation, hyperosmolar therapy was initiated with IV mannitol and hypertonic saline infusions. Decline in renal function with a fall in serum sodium to 131 mmol/L and minimal urine output was treated with continuous veno-venous hemofiltration. The patient’s neurologic status continued to decline with loss of brainstem reflexes. Given the patient’s likelihood of severe irreversible neurological disability, the possibility for successful liver transplantation was deemed unlikely and the patient’s family elected to withdraw care. After compassionate extubation, the patient expired on hospital day 3, less than 60 hours after his initial ED presentation.

## DISCUSSION

Acute liver failure is defined as severe acute liver injury, concurrent with encephalopathy and evidence for loss of hepatic synthesis, manifested by elevated prothrombin time or INR, in a patient without cirrhosis or pre-existing liver disease.^1^ Acute liver failure can be further subcategorized by the time course of illness, whereby hyperacute disease is defined as illness duration less than seven days, acute disease 7–21 days, and subacute disease greater than 21 days. Cerebral edema is more common in hyperacute and acute disease.^5^

Evaluation of acute liver failure in the ED should focus on identification of treatable causes. Etiologies include viruses, hepatotoxic drugs (most commonly acetaminophen), amanita mushroom poisoning, idiosyncratic drug reactions, autoimmune hepatitis, environmental toxins, complications of pregnancy, sepsis, malignancy, and vascular conditions including acute Budd-Chiari syndrome.^6^,^7^ The most common etiologies of acute liver failure in the U.S. are acetaminophen toxicity and idiosyncratic drug reactions; the low rate of viral hepatitis in developed countries is reflective of immunization programs, improved screening of blood products, and public health measures.^8^,^9^ The following table summarizes commonly reported causes of acute liver failure.^6^–^10^

Acute hepatitis B is usually subclinical with only 30% of cases resulting in clinically apparent icteric disease.^10^ Acute liver failure is a rare occurrence seen in only 0.1–0.5% of patients with acute hepatitis B.^11^ Possible risk factors for severe course of illness include co-ingestion of acetaminophen, alcohol, or amphetamine use during illness.^11^ Prodromal fever and temperature greater than 38 °C are independent risk factors for development of acute liver failure.^12^

The diagnosis of acute hepatitis B may not be apparent in the ED setting, as viral serologies generally do not have rapid turnaround time. Inferences may be drawn by patterns of abnormalities of liver enzymes, which may be interpreted as predominantly hepatocyte injury vs. biliary tract obstruction. Hepatocyte injury is suggested by a pattern of elevation in aspartate aminotransferase and alanine aminotransferase. Predominant elevations in alkaline phosphatase and γ-glutamyl-transpeptidase are suggestive of a cholestatic injury pattern.^13^

Antiviral therapies are mainstays of therapy for severe acute hepatitis B.^3^ The aims of antiviral therapy are to reverse or delay complications of cirrhosis and to decrease the risk of viral reinfection if patients ultimately receive a liver transplant. Lamivudine may be associated with higher rates of progression to chronic hepatitis B, not seen with entecavir.^14^ One study suggests that early treatment (within one week of illness onset) with lamivudine may lead to decreased mortality compared to both control cases and delayed treatment (greater than one week after onset of illness).^15^

*N*-acetylcysteine is well established in the treatment of acetaminophen toxicity. Its utility in cases of acute liver failure from other causes is under debate.^3^,^16^ *N*-acetylcysteine improves microcirculatory blood flow and oxygen delivery to vital organs by acting as an anti-inflammatory agent, antioxidant, and inotrope, and has improved transplant–free survival in one study of patients with non-acetaminophen-induced acute liver failure.^17^–^19^

Consideration should be given to treatment of coagulopathy related to acute liver failure. Current recommendations suggest a one-time prophylactic dose of 5–10 mg of Vitamin K1.^20^ Treatment with fresh frozen plasma in the absence of active bleeding is not indicated and carries a risk of exacerbating volume overload and causing transfusion-related acute lung injury.^1^

## CONCLUSION

Acute liver failure from acute hepatitis B is a rare but potentially lethal complication.^6^ The transplant-free survival rate in patients with acute hepatitis B is only 19%, and case fatalities are associated with hepatic encephalopathy and cerebral edema.^21^ Although viral causes of acute liver failure are uncommon, this case illustrates the importance of keeping viral hepatitis in the differential diagnosis for patients with acute liver failure. Early identification, directed therapy, and treatment at a transplantation center are associated with increased survival.^6^

Documented patient informed consent and/or Institutional Review Board approval has been obtained and filed for publication of this case report.

## Figures and Tables

**Table:** Etiologies of acute liver failure.

Etiologies of acute liver failure	Commonly reported	Uncommonly reported
Toxic/drug induced	Acetaminophen Ethanol Isoniazid Phenytoin Carbamazepine Valproate	Rifampicin Methotrexate Halogenated hydrocarbons *Amanita* mushroom
Viral	Hepatitis A,B,D,E	Hepatitis C Cytomegalovirus Epstein-Barr virus Herpes viruses Varicella zoster Parvo-virus B19 Adenovirus Hemorrhagic fevers Yellow fever
Herbal products		Black cohosh Chaparral Kava Camphor Castor oil
Vascular	Veno-occlusive disease Budd-Chiari Sepsis/“shock liver”	
Metabolic		Wilson disease Alpha-1-antitrypsin deficiency Acute fatty liver of pregnancy Reye syndrome
Miscellaneous		Non-alcoholic steatohepatitis Autoimmune hepatitis Heatstroke Malignant infiltration: lymphoma, leukemia
